# A Five-Component Biginelli-Diels-Alder Cascade Reaction

**DOI:** 10.3389/fchem.2018.00376

**Published:** 2018-08-24

**Authors:** Taber S. Maskrey, Madeline C. Frischling, Mikhaila L. Rice, Peter Wipf

**Affiliations:** Department of Chemistry, University of Pittsburgh, Pittsburgh, PA, United States

**Keywords:** Biginelli reaction, dihydropyrimidinone, multi-component condensation, 5-CC, hetero Diels-Alder reaction, InBr_3_

## Abstract

A new multi-component condensation was discovered during the reaction of a urea, β-keto ester, and formaldehyde. In the presence of catalytic indium bromide, a Biginelli dihydropyrimidinone intermediate was further converted to a five-component condensation product through a formal hetero Diels-Alder reaction. The product structure was confirmed by NMR and NOE analysis, and the proposed stepwise mechanism was supported by the reaction of the Biginelli intermediate with ethyl 2-methylene-3-oxobutanoate.

## Introduction

**Figure 1:**
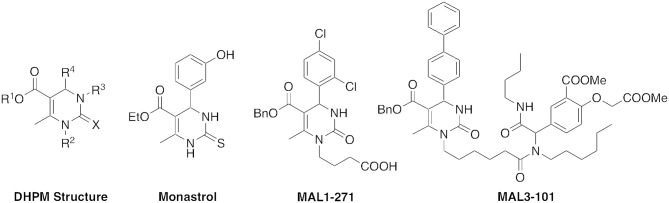
DHPM structure and biologically active analogs.

Dihydropyrimidinones (DHPMs) represent an attractive class of biologically active small molecules. DHPMs have been shown to have neuroprotective, antiparasitic, antiviral, antitumor, anti-inflammatory, and anticancer activities (de Fátima et al., 2015). Monastrol, for example, is one of the most studied DHPMs based on its antiproliferative activity (Figure 1). In 1999, monastrol was found to disrupt mitosis (Mayer et al., 1999; Kapoor et al., 2000). Subsequently, several potent analogs with increased anticancer potency have also been synthesized (Ragab et al., 2017).

The DHPM structure is the product of a reaction between a urea, β-keto ester, and an aldehyde, commonly termed the Biginelli reaction. The creation of a structurally diverse library of pharmacologically active compounds is an attractive possibility with this three-component condensation (Wan and Pan, 2012). As part of our studies on the preparation of bioactive DHPMs, in particular heat shock protein 70 (Hsp70) antagonists and agonists such as MAL3-101 and MAL1-271 (Figure 1), we explored the scope of Biginelli reactions in solution, fluorous media, and on solid support, and developed analogs with potent anticancer, antiviral, and neuroprotective properties (Wipf and Cunningham, 1995; Studer et al., 1997; Huryn et al., 2011; Manos-Turvey et al., 2016).

**Figure 2:**
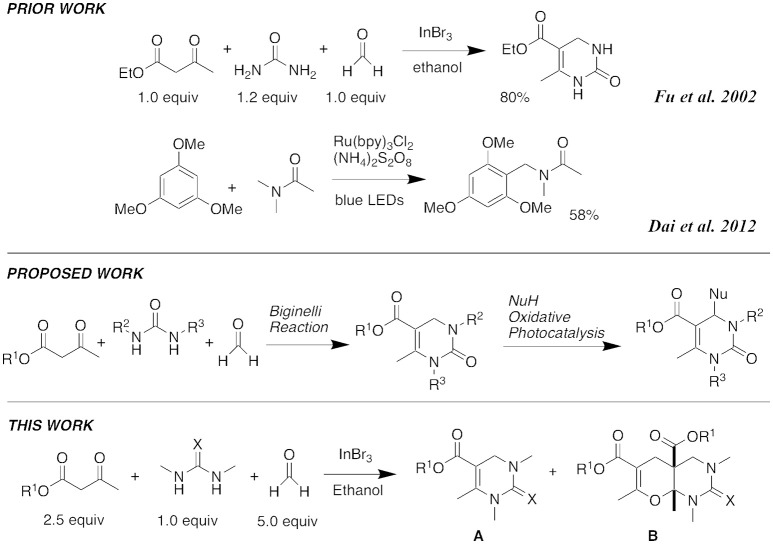
Literature precedence and synthetic plans.

Standard Biginelli reactions frequently use catalytic amounts of Brønsted acids, such as hydrochloric acid (HCl) (Nagarajaiah et al., 2016). Various Lewis acid catalysts, including InCl_3_ (Ranu et al., 2000), FeCl_3_ (Lu and Bai, 2002), BF_3_•OEt_2_ (Hu et al., 1998), ZnCl_2_ (Sun et al., 2004), GaCl_3_ (Yuan et al., 2017), and InBr_3_ have also been used successfully (Phucho et al., 2009). While working to advance the scope of our own DHPM library, we noted the use of 10% InBr_3_ as a catalyst at reflux in ethanol for 7 h to produce DHPMs (Fu et al., 2002; Martins et al., 2004). We decided to explore these reaction conditions further as they gave good yields even with formaldehyde as a reaction component. Initially, we hoped to be able to introduce substituents at the 4-position of the DHPM ring through an oxidative photochemical reaction related to the previously reported Friedel-Crafts amidoalkylation with the visible light catalyst, Ru(bpy)_3_Cl_2_ (Dai et al., 2012) (Figure 2). However, we found that InBr_3_ conditions promoted a new five-component condensation with formaldehyde, and decided to first investigate the scope of this process.

## Results and discussion

**Figure 3:**
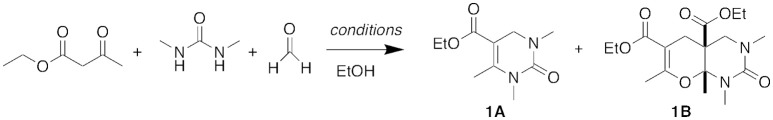
Five-component condensation reaction. For conditions, see Table 1.

The originally reported InBr_3_ reaction conditions utilized an excess of urea and equal equivalents of β-keto ester and aldehyde. For our initial test reactions, we chose *N,N*′-dimethylurea as the limiting reagent. The reaction of *N,N*′-dimethylurea, 1.8 equivalents of ethyl acetoacetate as the β-keto ester, and 3 equivalents of formaldehyde in 95% ethanol at reflux for 7 h yielded not only the expected DHPM product **1A** but also a higher molecular weight product that we did not anticipate (Figure 3). Through NMR and NOE analyses, we were able to assign its fused bicyclic structure as **1B**. We hypothesized that this secondary product occurred through a five-component condensation reaction, and an *in situ* formal hetero Diels-Alder reaction of the expected DHPM Biginelli intermediate was in agreement with the product structure.

Prior reports of the product of a Biginelli reaction being utilized as a dienophile in a hetero Diels-Alder reaction are rare. Sharma and coworkers demonstrated the reaction of an isolated Biginelli heterocycle with *N*-arylidine-*N*′-methylformamidines and *N*-arylidine guanidine in THF (Sharma et al., 2005). Our present synthesis represents the first one pot reaction to form these highly functionalized bicyclic products from readily available precursors.

We subsequently explored a range of reaction conditions in an attempt to improve overall yield and selectivity (Figure 3). For these optimizations, we used our original reaction components *N,N*′-dimethylurea, ethyl acetoacetate, and formaldehyde (Table 1). The traditional Brønsted acid catalyst, HCl, gave no conversion with formaldehyde (entry 2). We experimented with other Lewis acids (entries 3–6) to catalyze the reaction but saw no conversion with FeCl_3_ or ZnCl_2_. Conversion was modest with both InCl_3_ and AlCl_3_. Increasing the proportions of the β-keto ester and the aldehyde to 2.5 and 5 equivalents, respectively, improved the yield of both products **1A** and **1B** (entry 7). However, increasing or decreasing the reactant concentration (entries 8 and 9) did not improve the overall yield. Doubling the reaction time to 14.5 h also did not increase the conversion to product **1B** (entry 10 vs. entry 7). After a shortened reaction time of 2.0 h, only product **1A** was isolated in 28% yield (entry 11). Accordingly, entry 7 represented our optimized conditions: 1 equivalent of urea, 2.5 equivalents of β-keto ester, 5.0 equivalents of aldehyde, and 0.1 equivalents of InBr_3_ at reflux conditions in ethanol (0.2 M) for 7 h provided 45% of DHPM **1A** and 48% of pyranopyrimidinone **1B**.

**Table 1:** Investigation of reaction time, concentration, equivalents, and catalyst with formaldehyde, *N,N*′-dimethylurea, and ethyl acetoacetate (Figure 3).

**Entry**	**Urea (equiv)**	**Ester (equiv)**	**Aldehyde (equiv)**	**Catalyst (equiv)**	**Time (h)**	**Concentration (M)**	**A (% Yield)**	**B (% Yield)**
1	1	1.8	3	InBr_3_ (0.1)	7	0.2	10	26
2	1	1.8	3	HCl (1.0)	7	0.5	0	0
3	1	1.8	3	InCl_3_ (0.1)	7	0.3	23	25
4	1	1.8	3	AlCl_3_ (0.1)	7	0.3	3	14
5	1	1.8	3	FeCl_3_ (0.2)	7	0.2	0	0
6	1	1.8	3	ZnCl_2_ (0.2)	7	0.3	0	0
7	1	2.5	5	InBr_3_ (0.1)	7	0.2	45	48
8	1	2.5	5	InBr_3_ (0.1)	7	1.0	55	37
9	1	2.5	5	InBr_3_ (0.1)	7	0.05	38	27
10	1	2.5	5	InBr_3_ (0.1)	14.5	0.2	41	36
11	1	2.5	5	InBr_3_ (0.1)	2	0.2	28	0

**Figure 4:**
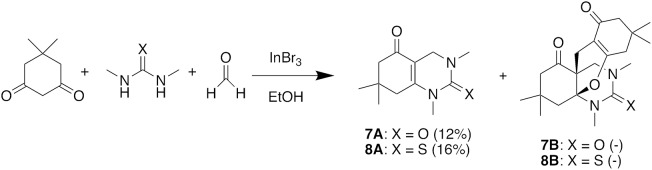
Attempted five-component condensation reaction with a cyclic 1,3-diketone.

We then used these optimized conditions to further explore the scope of the reaction (Table 2). Formaldehyde was used as the aldehyde component in all reactions since preliminary trials with other aldehydes and ketones were not successful in producing any fused bicycles **B**. We also used symmetrical ureas exclusively to avoid possible regioisomers (exploratory reactions confirmed a lack of regioselectivity with unsymmetrical ureas; for example, while 1-methylurea and 1-(4-methoxyphenyl)-3-methylurea provided both the Biginelli DHPM and the Diels-Alder products, we were unable to separate the regioisomers formed in an approximately 1:1 ratio). Curiously, the reaction with the *N,N*′-dimethylthiourea stopped at the Biginelli product **2A**, which was isolated in 61% yield (entry 2). Methyl acetoacetate with thiourea **2** (entry 3) also gave similar results, but product **3A** was formed in lower yield, likely due to *trans*-esterification with ethanol. Utilizing benzyl acetoacetate, we were able to isolate both Biginelli (**4A**, **5A**) and five-component condensation products (**4B**, **5B**) in good overall yields with the urea and thiourea derivatives (entries 4 and 5). However, the yield of the five-component condensation Diels-Alder product **4B** was considerably higher with the urea derivative than the pyranopyrimidinethione **5B** resulting from the thiourea. With allyl acetoacetate and *N,N*′-dimethylurea, we were able to isolate the five-component condensation product **6B**, but not the corresponding Biginelli intermediate, as it proved unstable. We also attempted to replace the traditional β-keto ester with 5,5-dimethyl-1,3-cyclohexanedione (dimedone) to probe the feasibility of adding an additional ring in the hetero Diels-Alder reaction (Figure 4). Unfortunately, with both *N,N*′-dimethylurea and its thiourea equivalent, we were only able to isolate Biginelli products **7A** and **8A**, in significantly lower yields than for the β-keto ester conversions.

**Table 2:** Reaction scope with formaldehyde, *N,N*′-dimethyl(thio)urea (X=O,S), and alkyl (R^1^) acetoacetates. For products A and B, see Figure 2.

**No**.	**R^1^**	**X**	**A (% Yield)**	**B (% Yield)**
1	Ethyl	O	45	48
2	Ethyl	S	61	0
3	Methyl	S	31	0
4	Benzyl	O	12	73
5	Benzyl	S	61	32
6	Allyl	O	0	21

**Figure 5:**
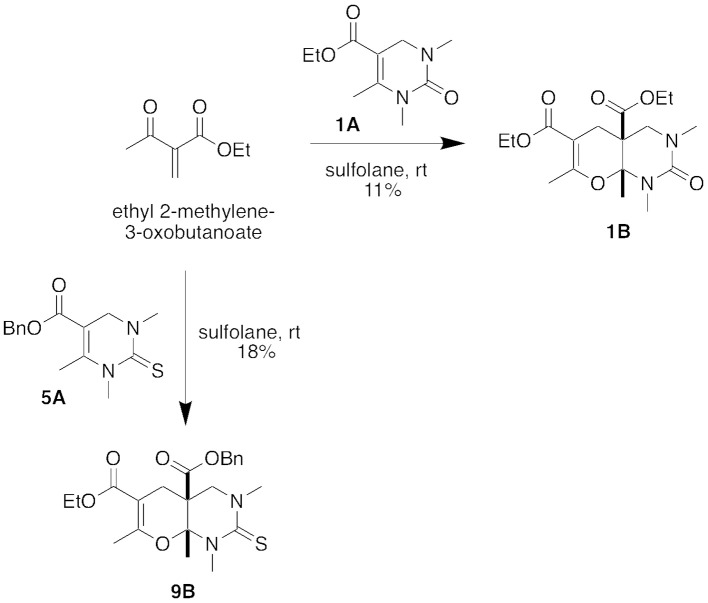
Stepwise conversions of intermediate DHPM (Biginelli) products in a hetero Diels-Alder reaction.

To confirm our hypothesis that the five-component condensation product formed through a hetero Diels-Alder reaction with the DHPM (Biginelli) intermediate, we reacted Biginelli product **1A** with ethyl 2-methylene-3-oxobutanoate (Figure 5). After stirring in sulfolane at room temperature for 18 h, we isolated the corresponding hetero Diels-Alder Product, **1B**, in 11% yield. We were also able to repeat this conversion with the Biginelli intermediate **5A** to form the hetero Diels-Alder Product **9B**.

**Figure 6:**
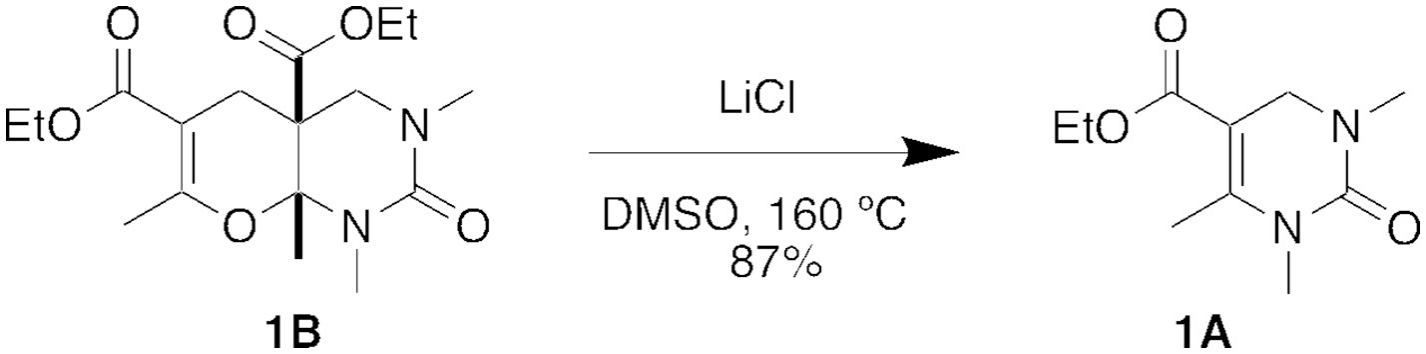
Retro Diels-Alder reaction under Krapcho dealkylation conditions.

Interestingly, when the five-component condensation product **1B** was subjected to Krapcho dealkylation conditions with LiCl in DMSO, we isolated the DHPM product, **1A**, in 87% yield, presumable through a retro Diels-Alder reaction (Figure 6) (Krapcho et al., 1967).

**Figure 7:**
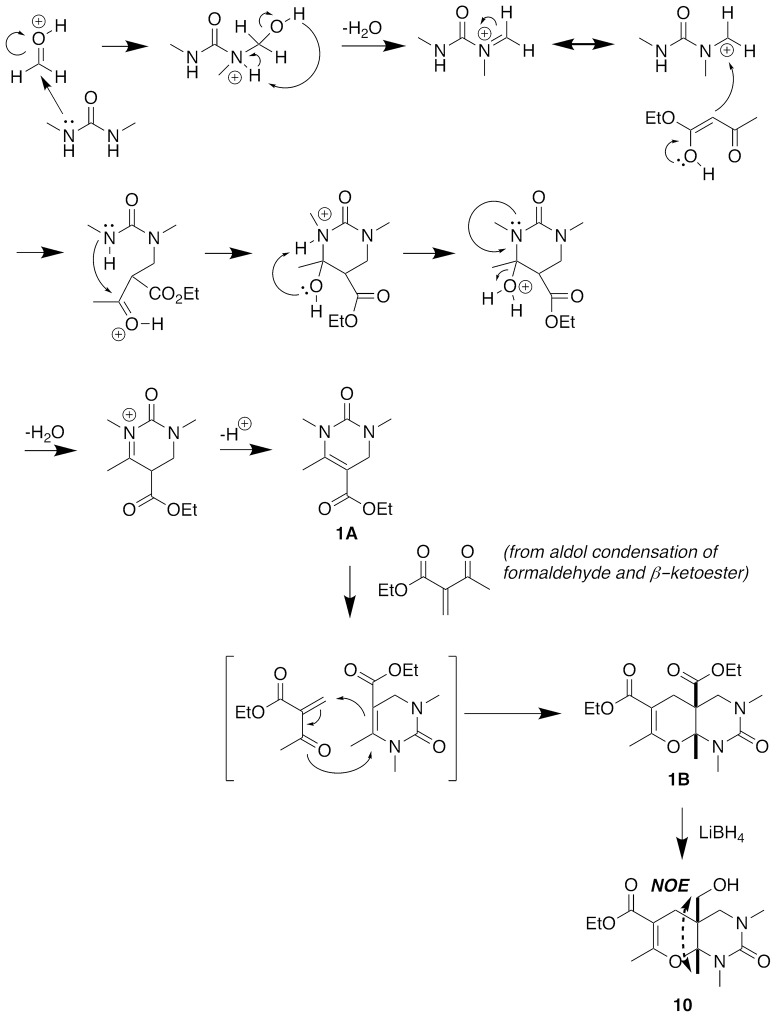
Proposed mechanism of five-component condensation.

Figure 7 illustrates our proposed mechanism for the five-component condensation reaction. In the presence of a Lewis acid, condensation of the urea with the aldehyde and subsequent loss of water generates a sufficiently reactive electrophile for attack by the β-keto ester, which then undergoes cyclization to give **1A**. The excess β-keto ester and formaldehyde react to form a methylene group at the α-position, and this electrophile then undergoes a hetero Diels-Alder reaction with the intermediate DHPM **1A** to provide the fused heterocyclic **1B** as a single stereoisomer. We determined the configuration of the methyl group to be *cis* to the ester at the two quaternary ring fusion atoms by converting the ester to the primary alcohol **10** with LiBH_4_, and then using a NOE analysis to confirm that the methyl group protons showed a >10% percent double resonance enhancement with the newly formed CH_2_ protons.

## Conclusions

We have discovered and optimized experimental conditions for a novel one pot, five-component condensation reaction with a β-keto ester, urea, and formaldehyde. The reaction appears to proceed through an intermediate DHPM (Biginelli) product. The DHPM then reacts with the condensation product of the β-keto ester and formaldehyde through a formal hetero Diels-Alder reaction. While the scope of this new process is still limited to formaldehyde and symmetrical *N,N*′-dialkylated ureas, it provides easy access to bicyclic ring systems that were previously inaccessible through a single transformation.

## Materials and methods

### General

All reagents were obtained commercially unless otherwise noted. All glassware was dried in an oven at 140°C for 2 h prior to use. Reactions were monitored by TLC analysis (EMD Millipore pre-coated silica gel 60 F254 plates, 250 μM layer thickness) and visualization was accomplished with a 254 nM UV light or staining with a PMA solution (5 g of phosphomolybdic acid in 100 mL of 95% EtOH), p-anisaldehyde solution (2.5 mL of p-anisaldehyde, 2 mL of AcOH, and 3.5 mL of conc. H_2_SO_4_ in 100 mL of 95% EtOH), or a KMnO_4_ solution (1.5 g of KMnO_4_ and 1.5 g of K_2_CO_3_ in 100 mL of a 0.1% NaOH solution). Some purification by chromatography was performed using a SiO_2_ Büchi flash chromatography system. ^1^H/^13^C NMR spectra were recorded on either a Bruker Avance 300/75 MHz, Bruker Avance 400/100 MHz, Bruker Avance 500/135 MHz, or Bruker Avance 600/150 MHz instruments. Chemical shifts were reported in parts per million with the residual solvent peak used as the internal standard (^1^H/^13^C: CDCl_3_, 7.26, 77.0 ppm; CD_3_OD, 3.31, 49.3 ppm; DMSO, 2.50, 39.5 ppm). Chemical shifts were tabulated as follows: chemical shift, multiplicity (s = singlet, d = doublet, t = triplet, q = quarter, dd = doublet of doublet, dt = doublet of triplet, dq = doublet of quartet, m = multiplet, b = broad, app = apparent), coupling constants, and integration. All 1D NMR spectra were processed using Bruker Topspin NMR. IR spectra were obtained on an Identity IR-ATR spectrometer. Melting points (uncorrected) were determined using a Mel-Temp instrument. HRMS data were obtained on a Thermo Scientific Exactive HRMS coupled to a Thermo Scientific Accela HPLC system using a 2.1 x 50 mm 3.5 μm Waters XTerra C18 column eluting with MeCN/H_2_O containing 0.1% formic acid.

### General 5-component condensation reaction procedure

Representative procedure for **ethyl 1,3,6-trimethyl-2-oxo-1,2,3,4-tetrahydropyrimidine-5-carboxylate (1A) and diethyl(4a*SR*,8a*RS*)-1,3,7,8a-tetramethyl-2-oxo-1,3,4,8a-tetrahydro-2*H*-pyrano[2,3-*d*]pyrimidine-4a,6(5*H*)-dicarboxylate (1B)**. A solution of ethyl acetoacetate (1.21 mL, 9.43 mmol, 2.5 equiv), paraformaldehyde (0.478 g, 15.3 mmol, 5 equiv), and *N,N*′-dimethylurea (0.456 g, 5.07 mmol, 1 equiv) in 95% ethanol (25 mL) was stirred at room temperature for 10 min. Indium(III) bromide (0.180 g, 0.507 mmol, 0.1 equiv) was added and the reaction mixture was heated under reflux for 7 h, cooled to room temperature, filtered through basic Al_2_O_3_ (EtOAc) and concentrated under reduced pressure. The residue was purified by chromatography on SiO_2_ (hexanes/EtOAc, 3:1 to 1:3) to afford **1A** (0.481 g, 2.26 mmol, 45%) as a light yellow wax and **1B** (0.855 g, 2.41 mmol, 48%) as a yellow oil. This procedure was followed for all products in Table 2 and Figure 4, and spectral properties are presented below.

**Ethyl 1,3,6-trimethyl-2-oxo-1,2,3,4-tetrahydropyrimidine-5-carboxylate (1A)**. IR (ATR) 2981.1, 2908.3, 1672.9, 1622.7, 1414.1, 1262.5, 1204.0, 1077.4, 1026.8, 749.3 cm^−1^; ^1^H NMR (400 MHz, CD_3_OD) δ 4.16 (q, *J* = 7.1 Hz, 2 H), 4.01 (d, *J* = 1.1 Hz, 2 H), 3.17 (s, 3 H), 2.92 (s, 3 H), 2.46 (t, *J* = 1.3 Hz, 3 H), 1.28 (t, *J* = 7.1 Hz, 3 H); ^13^C NMR (100 MHz, CD_3_OD) δ 165.9, 154.8, 150.8, 98.3, 59.8, 48.5, 34.2, 29.8, 14.8, 13.2; HRMS (HESI) *m/z* calcd for C_10_H_17_N_2_O_3_ [M+H]^+^ 213.1234, found 213.1234.

**Diethyl(4a*SR*,8a*RS*)-1,3,7,8a-tetramethyl-2-oxo-1,3,4,8a-tetrahydro-2*H*-pyrano[2,3-*d*]pyrimidine-4a,6(5*H*)-dicarboxylate (1B)**. IR (ATR) 2987.8, 2931.8, 1705.4, 1629.0, 1498.5, 1442.6, 1343.8, 1233.9, 1086.6, 1041.9, 984.1, 870.4, 844.3, 753.0 cm^−1^; ^1^H NMR (400 MHz, CD_3_OD) δ 4.22–4.14 (m, 4 H), 3.53 (d, *J* = 12.7 Hz, 1 H), 3.30 (d, *J* = 12.7 Hz, 1 H), 2.95 (s, 3 H), 2.92 (s, 3 H), 2.69 (dq, *J* = 17.8, 1.7 Hz, 1 H), 2.45 (dq, *J* = 17.8, 1.6 Hz, 1 H), 2.24 (t, *J* = 1.6 Hz, 3 H), 1.61 (s, 3 H), 1.28 (t, *J* = 7.2 Hz, 3 H), 1.25 (t, *J* = 7.2 Hz, 3 H); ^13^C NMR (100 MHz, CD_3_OD) δ 170.6, 167.2, 160.2, 154.9, 99.8, 87.8, 61.4, 59.9, 49.3, 44.0, 34.9, 27.6, 27.5, 19.5, 18.5, 13.5, 13.1; HRMS (HESI) *m/z* calcd for C_17_H_27_N_6_O_2_ [M+H]^+^ 355.1864, found 355.1862.

**Ethyl 1,3,6-trimethyl-2-thioxo-1,2,3,4-tetrahydropyrimidine-5-carboxylate (2A)**. IR (ATR) 2978.4, 1670.0, 1636.5, 1524.6, 1442.6, 1356.9, 1267.4, 1207.8, 1168.6, 1140.7, 1088.5, 1015.8 cm^−1^; ^1^H NMR (400 MHz, CDCl_3_) δ 4.20 (q, *J* = 7.1 Hz, 2 H), 4.06 (d, *J* = 1.0 Hz, 2 H), 3.55 (s, 3 H), 3.42 (s, 3 H), 2.45 (t, *J* = 1.0 Hz, 3 H), 1.30 (t, *J* = 7.1 Hz, 3 H); ^13^C NMR (100 MHz, CDCl_3_) δ 180.8, 165.3, 149.2, 101.3, 60.5, 47.9, 42.9, 37.9, 16.2, 14.3; HRMS (HESI) *m/z* calcd for C_10_H_17_N_2_O_2_S [M+H]^+^ 229.1005, found 229.1004.

**Methyl 1,3,6-trimethyl-2-thioxo-1,2,3,4-tetrahydropyrimidine-5-carboxylate (3A)**. IR(ATR) 2929.7, 2828.8, 1698.0, 1619.2, 1516.3, 1459.9, 1425.5, 1366.5, 1317.3, 1261.1, 1169.4, 1091.3, 990.9, 803.2, 765.3 cm^−1^; ^1^H-NMR (400 MHz, CDCl_3_) δ 4.09 (s, 2 H), 3.77 (s, 3 H), 3.58 (s, 3 H), 3.45 (s, 3 H), 2.48 (s, 3 H); ^13^C NMR (100 MHz, CDCl_3_) δ 180.8, 165.7, 149.6, 101.0, 51.6, 47.9, 42.9, 37.9, 16.3; HRMS (HESI) *m/z* calcd for C_9_H_15_N_2_O_2_S [M+H]^+^ 215.0849, found 215.0848.

**Benzyl 1,3,6-trimethyl-2-oxo-1,2,3,4-tetrahydropyrimidine-5-carboxylate (4A)**. IR (ATR) 2933.0, 1662.6, 1554.5, 1485.5, 1450.1, 1192.9, 1127.6, 1077.3, 1027.0, 691.5 cm^−1^; ^1^H-NMR (300 MHz, CD_3_OD) δ 7.37–7.34 (m, 5 H), 5.17 (s, 2 H), 4.04 (bd, *J* = 1.2 Hz, 2 H), 3.17 (s, 3 H), 2.91 (s, 3 H), 2.47 (t, *J* = 1.2 Hz, 3 H); ^13^C NMR (75 MHz, CDCl_3_) δ 180.8, 165.0, 149.9, 136.0, 128.6, 128.3, 128.1, 101.8, 66.3, 47.9, 42.9, 37.9, 16.4; HRMS (HESI) *m/z* calcd for C_15_H_19_N_2_O_3_ [M+H]^+^ 275.1390, found 275.1388.

**Dibenzyl(4a*SR*,8a*RS*)-1,3,7,8a-tetramethyl-2-oxo-1,3,4,8a-tetrahydro-2*H*-pyrano[2,3-*d*]pyrimidine-4a,6(5*H*)-dicarboxylate (4B)**. IR (ATR) 2937.4, 2881.5, 1712.9, 1629.0, 1506.0, 1448.2, 1379.3, 1347.6, 1232.0, 1077.3, 982.3, 883.5, 756.7 cm^−1^; ^1^H NMR (300 MHz, CD_3_OD) δ 7.35–7.32 (m, 10 H), 5.19, 5.15 (AB, *J* = 16.4 Hz, 2 H), 5.15 (s, 2 H), 3.54 (d, *J* = 12.9 Hz, 1 H), 3.27 (d, *J* = 12.9 Hz, 1 H), 2.90 (s, 3 H), 2.84 (s, 3 H), 2.73 (dq, *J* = 17.8, 1.6 Hz, 1 H), 2.48 (dq, *J* = 17.8, 1.6 Hz, 1 H), 2.20 (t, *J* = 1.6 Hz, 3 H), 1.54 (s, 3 H); ^13^C NMR (100 MHz, CDCl_3_) δ 170.6, 167.0, 161.2, 154.1, 136.2, 135.0, 128.6, 128.5 (2C), 128.1 (2C), 128.0, 99.6, 87.6, 67.3, 66.0, 49.7, 44.1, 35.7, 28.1, 27.8, 20.7, 19.7; HRMS (HESI) *m/z* calcd for C_27_H_31_N_2_O_6_ [M+H]^+^ 479.2177, found 479.2176.

**Benzyl 1,3,6-trimethyl-2-thioxo-1,2,3,4-tetrahydropyrimidine-5-carboxylate (5A)**. IR (ATR) 2933.7, 1712.9, 1629.0 1450.1, 1358.8, 1254.4, 1215.2, 1163.0, 1094.1, 1066.1, 1028.8, 734.4 cm^−1^; ^1^H-NMR (600 MHz, CDCl_3_) δ 7.39–7.32 (m, 5 H), 5.19 (s, 2 H), 4.08 (d, *J* = 1.0 Hz, 2 H), 3.55 (s, 3 H), 3.41 (s, 3 H), 2.47 (d, *J* = 1.0 Hz, 3 H); ^13^C NMR (150 MHz, CDCl_3_) δ 180.8, 165.0, 149.9, 136.0, 128.6, 128.30, 128.17, 100.9, 66.3, 47.9, 43.0, 38.0, 16.4; HRMS (HESI) *m/z* calcd for C_15_H_19_N_2_O_2_S [M+H]^+^ 291.1162, found 291.1160.

**Dibenzyl(4a*SR*,8a*RS*)-1,3,7,8a-tetramethyl-2-thioxo-1,3,4,8a-tetrahydro-2*H*-pyrano[2,3-*d*]pyrimidine-4a,6(5*H*)-dicarboxylate (5B)**. IR (ATR) 2933.7, 1712.9, 1629.0, 1450.1, 1340.1, 1232.0, 1215.2, 1077.3, 1002.8, 995.3, 734.4, 695.2 cm^−1^; ^1^H NMR (300 MHz, CDCl_3_) δ 7.35–7.32 (m, 10 H), 5.19–5.08 (m, 4 H), 3.79 (d, *J* = 13.6 Hz, 1 H), 3.39 (s, 3 H), 3.34 (s, 3 H), 3.31 (d, *J* = 13.5 Hz, 1H), 2.63 (dd, *J* = 17.8, 1.5 Hz, 1 H), 2.36 (dd, *J* = 17.8, 1.5 Hz, 1 H), 2.23 (t, *J* = 1.5 Hz, 3 H), 1.51 (s, 3 H); ^13^C NMR (100 MHz, CD_3_OD) δ 178.3, 169.0, 166.8, 160.1, 136.4, 135.4, 128.3, 128.2 (2C), 128.1 (2C), 128.0, 127.9 (2C), 127.8, 101.1, 86.5, 68.0, 67.1, 65.7, 51.5, 43.1, 42.7, 34.1, 26.0, 20.2, 18.1, 13.9; HRMS (HESI) *m/z* calcd for C_27_H_31_N_2_O_5_S [M+H]^+^ 495.1948, found 495.1945.

**Diallyl(4a*SR*,8a*RS*)-1,3,7,8a-tetramethyl-2-oxo-1,3,4,8a-tetrahydro-2*H*-pyrano[2,3-*d*]pyrimidine-4a,6(5*H*)-dicarboxylate (6B)**. IR (ATR) 2934.3, 1728.4, 1709.3, 1646.5, 1500.4, 1444.8, 1410.4, 1345.0, 1234.1, 1213.6, 1102.4, 1081.7, 1039.7, 986.7, 881.9, 753.5 cm^−1^; ^1^H NMR (400 MHz, DMSO-*d6*) δ 6.00–5.84 (m, 2 H), 5.32–5.21 (m, 4 H), 4.61 (dd, *J* = 16.0 Hz, 4.0 Hz, 4 H), 3.51 (d, *J* = 12.4 Hz, 1 H), 3.22 (d, *J* = 12.4 Hz, 1 H), 2.80 (s, 3 H), 2.68 (s, 3 H), 2.66 (d, *J* = 17.6 Hz, 1 H), 2.40 (d, *J* = 17.6 Hz, 1 H), 2.20 (s, 3 H), 1.52 (s, 3 H); ^13^C NMR (100 MHz, DMSO-*d6*) δ 170.5, 166.6, 160.6, 153.8, 133.5, 132.5, 118.1, 118.0, 99.9, 88.1, 65.8, 64.7, 49.3, 44.2, 35.7, 28.3, 27.7, 20.7, 19.7; HRMS (HESI) *m/z* calcd for C_19_H_27_N_2_O_6_ [M+H]^+^ 379.1864, found 379.1862.

**1,3,7,7-Tetramethyl-4,6,7,8-tetrahydroquinazoline-2,5(1*H*,3*H*)-dione (7A)**. IR (ATR) 2960.4, 2890.4, 1722.3, 1620.5, 1574.3, 1438.2, 1380.7, 1268.3, 1224.5, 1127.4, 1079.6, 1006.6 cm^−1^; ^1^H NMR (400 MHz, CDCl_3_) δ 4.04 (s, 2 H), 3.20 (s, 3 H), 2.98 (s, 3 H), 2.39 (s, 2 H), 2.24 (s, 2 H), 1.11 (s, 6 H); ^13^C NMR (100 MHz, CDCl_3_) δ 194.3, 153.5, 153.2, 105.6, 49.2, 45.6, 40.2, 35.8, 32.8, 30.4, 28.6; HRMS (HESI) *m/z* calcd for C_12_H_19_N_2_O_2_ [M+H]^+^ 223.1441, found 223.1440.

**1,3,7,7-Tetramethyl-2-thioxo-2,3,4,6,7,8-hexahydroquinazolin-5(1*H*)-one (8A)**. IR (ATR) 2950.5, 2922.5, 2868.5, 1724.1, 1629.0, 1450.1, 1261.8, 1215.2, 1127.6, 1064.3, 982.3 cm^−1^; ^1^H NMR (400 MHz, CDCl_3_) δ 4.12 (s, 2 H), 3.60 (s, 3 H), 3.44 (s, 3 H), 2.43 (s, 2 H), 2.26 (s, 2 H), 1.11 (s, 6 H); ^13^C NMR (100 MHz, CDCl_3_) δ 194.6, 180.1, 151.0, 107.4, 49.3, 45.8, 43.8, 40.6, 37.4, 33.1, 28.6; HRMS (HESI) *m/z* calcd for C_12_H_19_N_2_OS [M+H]^+^ 239.1213, found 239.1211.

### Diels-Alder reaction of DHPM intermediate to form 5-component condensation product:

**Ethyl 2-methyl-3-oxo-2-(phenylthio)butanoate**. A stirred solution of N-chlorosuccinimide (6.21 g, 45.1 mmol) in CH_2_Cl_2_ (66 mL) was treated with thiophenol (0.40 mL, 3.8 mmol) and heated to reflux. After the reaction mixture changed color from yellow to orange, indicating the initiation of the reaction, additional thiophenol (4.24 mL, 41.2 mmol) was added dropwise to maintain a gentle reflux. The mixture was then allowed to cool to RT. After 1 h, the mixture was further cooled to 4°C on an ice bath, and ethyl 2-methylacetoacetate (6.81 mL, 46.7 mmol) was added over a 30 min period. After warming to RT and stirring for an additional 30 min, HCl was removed by bubbling nitrogen through the product solution and the solvent was removed under reduced pressure. The residue was suspended in petroleum ether (44 mL) and filtered. The resulting solid filtrate was washed with 5 portions (44 mL each) of petroleum ether. The combined organic fractions were combined and concentrated to give ethyl 2-methyl-3-oxo-2-(phenylthio)butanoate (11.6 g, 46.1 mmol) as a dark yellow liquid in quantitative yield with a small impurity. This product was used for the next step without further purification: ^1^H NMR (400 MHz, CDCl_3_) δ 7.51–7.30 (m, 5 H), 4.28–4.19 (m, 2 H), 2.37 (s, 3 H), 1.49 (s, 3 H), 1.32–1.26 (m, 3 H).

**Ethyl 2-methyl-3-oxo-2-(phenylsulfinyl)butanoate**. A solution of MCPBA (4.89 g, 19.8 mmol) in CH_2_Cl_2_ (51 mL) was added dropwise to a cold (0°C) solution of ethyl 2-methyl-3-oxo-2-(phenylthio)butanoate (5.01 g, 19.9 mmol) in CH_2_Cl_2_ (51 mL). The reaction mixture was stirred for 50 min, filtered through a plug of neutral Al_2_O_3_ (CH_2_Cl_2_), and concentrated under reduced pressure to yield ethyl 2-methyl-3-oxo-2-(phenylsulfinyl)butanoate (5.31 g, 19.8 mmol, 98%) as a yellow liquid that was used for the next step without further purification: IR (ATR) 1,030 cm^−1^.

**Ethyl 2-methylene-3-oxobutanoate**. A solution of ethyl 2-methyl-3-oxo-2-(phenylsulfinyl)butanoate (5.21 g, 19.4 mmol) in sulfolane (8 mL) was heated at 60°C and 0.1 mm Hg in a Kugelrohr distillation setup for 2 h. The collection vial was cooled to −78°C in a dry ice bath. The tube was vented with nitrogen gas, and the first distillate of ethyl 2-methylene-3-oxobutanoate was collected as a colorless liquid (0.66 g). The distillation was repeated with the remaining solution at 90°C for 1 h and 110°C for 1 h to give a second batch of ethyl 2-methylene-3-oxobutanoate together with residual sulfolane (1.22 g), for an overall yield of 68% (1.87 g, 13.2 mmol). Characteristic signals for ethyl 2-methylene-3-oxobutanoate: ^1^H NMR (300 MHz, CDCl_3_) δ 6.44 (d, *J* = 0.9 Hz, 1 H), 6.41 (d, *J* = 0.9 Hz, 1 H), 4.30 (q, *J* = 7.2 Hz, 2 H), 2.43 (s, 3 H), 1.33 (t, *J* = 7.2 Hz, 3 H).

**(Diethyl(4a*SR*,8a*RS*)-1,3,7,8a-tetramethyl-2-oxo-1,3,4,8a-tetrahydro-2*H*-pyrano[2,3-*d*]pyrimidine-4a,6(5*H*)-dicarboxylate (1B)**. Ethyl 1,3,6-trimethyl-2-oxo-1,2,3,4-tetrahydropyrimidine-5-carboxylate (**1A**, 0.096 g, 0.45 mmol) was added to ethyl 2-methylene-3-oxobutanoate containing residual sulfolane (0.244 g, 1.72 mmol). The reaction mixture was stirred at room temperature for 18 h and then concentrated under reduced pressure. The residue was purified by gradient chromatography on SiO_2_ (hexanes/EtOAc, 4:1 to 1:1) to afford **1B** (0.0682 g, 0.192 mmol, 11%) as a yellow oil that was spectroscopically identical to **1B** previously obtained in the one-pot Biginelli-Diels-Alder reaction.

**4a-Benzyl 6-ethyl (4a*SR*,8a*RS*)-1,3,7,8a-tetramethyl-2-thioxo-1,3,4,8a-tetrahydro-2*H*-pyrano[2,3-*d*]pyrimidine-4a,6(5*H*)-dicarboxylate (9B)**. Benzyl 1,3,6-trimethyl-2-thioxo-1,2,3,4-tetrahydropyrimidine-5-carboxylate (**5A**, 0.043 g, 0.15 mmol) was added to ethyl 2-methylene-3-oxobutanoate containing residual sulfolane (0.021 g, 0.15 mmol). The mixture was stirred at room temperature for 18 h. Another 5 equivalents of ethyl 2-methylene-3-oxobutanoate containing residual sulfolane (0.105 g, 0.738 mmol) was added and the reaction mixture was stirred at room temperature for an additional 48 h. The mixture was concentrated under reduced pressure and the resulting residue was purified by chromatography on SiO_2_ (hexanes/EtOAc, 4:1 to 2:1) to afford **9B** (0.0113 g, 0.0261 mmol, 18%) as a yellow oil: IR (ATR) 2974.7, 2933.7, 1705.4, 1643.9, 1524.6, 1450.1, 1340.1, 1232.0, 1077.3, 734.4, 700.8 cm^−1^; ^1^H NMR (400 MHz, CDCl_3_) δ 7.34–7.25 (m, 5 H), 5.15, 5.12 (AB, *J* = 12.0 Hz, 2 H), 4.19–4.08 (m, 2 H), 3.78 (d, *J* = 13.6 Hz, 1 H), 3.38 (s, 3 H), 3.32–3.28 (m, 4 H), 2.60–2.55 (m, 1 H), 2.35–2.29 (m, 1 H), 2.20 (t, *J* = 1.6 Hz, 3 H), 1.48 (s, 3 H), 1.27–1.23 (m, 3 H); ^13^C NMR (100 MHz, CDCl3): δ 178.5, 170.0, 166.9, 159.6, 134.9, 128.7, 128.6, 128.1, 101.3, 86.1, 67.6, 61.7, 60.3, 52.2, 43.9, 43.1, 34.9, 21.5, 19.2, 14.3, 14.0; HRMS (HESI) *m/z* calcd for C_22_H_29_N_2_O_5_S [M+H]^+^ 433.1792, found 433.1788.

### Conversion of 5-component condensation product to DHPM under Krapcho conditions

**Ethyl 1,3,6-trimethyl-2-oxo-1,2,3,4-tetrahydropyrimidine-5-carboxylate (1A)**. A mixture of diethyl (4a*SR*,8a*RS*)-1,3,7,8a-tetramethyl-2-oxo-1,3,4,8a-tetrahydro-2*H*-pyrano[2,3-*d*]pyrimidine-4a,6(5*H*)-dicarboxylate (**1B**, 0.050 g, 0.14 mmol), LiCl (0.016 g, 0.37 mmol), DMSO (2 mL), and water (4 drops) was heated at 160°C and the reaction was monitored by TLC analysis (2:1, hexanes:EtOAc). After 4 h, the reaction mixture was cooled to room temperature and extracted with EtOAc. The organic layer was dried (MgSO_4_), filtered, and concentrated to yield ethyl 1,3,6-trimethyl-2-oxo-1,2,3,4-tetrahydropyrimidine-5-carboxylate (**1A**, 0.026 g, 0.12 mmol, 87%) as a light yellow oil that was spectroscopically identical to **1A** previously obtained in the one-pot Biginelli-Diels-Alder reaction.

### Structural confirmation by conversion to alcohol

**Ethyl (4a*RS*,8a*RS*)-4a-(hydroxymethyl)-1,3,7,8a-tetramethyl-2-oxo-1,3,4,4a,5,8a-hexahydro-2*H*-pyrano[2,3-*d*]pyrimidine-6-carboxylate (10)**. A mixture of 4 M LiBH_4_ in THF (0.16 mL, 0.63 mmol) and diethyl (4a*SR*,8a*RS*)-1,3,7,8a-tetramethyl-2-oxo-1,3,4,8a-tetrahydro-2*H*-pyrano[2,3-*d*]pyrimidine-4a,6(5*H*)-dicarboxylate (**1B**, 150 mg, 0.42 mmol) in Et_2_O (2.1 mL) was heated at 35°C for 3 h. The reaction was monitored by TLC analysis (2:1, EtOAc:hexanes). After 3 h, the reaction mixture was quenched with 1 M HCl with ice-cooling. The solution was diluted with water and extracted with CH_2_Cl_2_ (3 × 5 mL). The organic layer was dried (MgSO_4_), filtered, and concentrated under reduced pressure to yield **10** (0.0794 g, 0.254 mmol, 60%) as white solid: Mp 114.8–116.9°C; IR (ATR) 3367.1, 2923.7, 1702.4, 1621.6, 1506.7, 1445.3, 1406.9, 1377.5, 1346.8, 1291.2, 1250.2, 1159.3, 1110.5, 985.0, 884.9, 838.9, 753.7 cm^−1^; ^1^H NMR (400 MHz, DMSO) δ 5.01 (t, *J* = 5.4 Hz, 1 H), 4.08 (q, *J* = 7.2 Hz, 2 H), 3.42 (dd, *J* = 10.8, 5.2 Hz, 1 H), 3.27 (dd, *J* = 10.8, 5.2 Hz, 1 H), 3.12 (d, *J* = 12.0 Hz, 1 H), 2.94 (d, *J* = 12.0 Hz, 1 H), 2.81 (s, 3 H), 2.80 (s, 3 H), 2.32 (bd, *J* = 16.4 Hz, 1 H), 2.15 (s, 3 H), 2.13 (bd, *J* = 16.4 Hz, 1 H), 1.37 (s, 3 H), 1.21 (t, *J* = 7.2 Hz, 3 H); ^13^C NMR (100 MHz, DMSO) δ 167.6, 159.9, 154.3, 100.0, 89.9, 62.1, 59.9, 48.7, 37.6, 35.9, 28.1, 27.1, 19.8, 19.7, 14.8; HRMS (HESI) *m/z* calcd for C_15_H_25_N_2_O_5_ [M+H]^+^ 313.1758, found 313.1759.

## Author contributions

TM, MF, and MR worked on the presented work under the guidance of PW. The manuscript was written by TM and PW with input from all authors.

### Conflict of interest statement

The authors declare that the research was conducted in the absence of any commercial or financial relationships that could be construed as a potential conflict of interest.
